# ATP metabolism in skeletal muscle arterioles

**DOI:** 10.1002/phy2.207

**Published:** 2014-01-28

**Authors:** Audrey J. Stone, Kirk W. Evanson, Heidi A. Kluess

**Affiliations:** 1 Department of Health Science, Kinesiology, Recreation and Dance, University of Arkansas, Fayetteville, Arkansas; 2 Pennsylvania State University College of Medicine, Hershey, Pennsylvania; 3 School of Kinesiology, Auburn University, Auburn, Alabama

**Keywords:** Aging, development, ecto‐ATPase, neurotransmitter

## Abstract

The purpose of this study was to investigate the metabolism of Adenosine triphosphate (ATP) in skeletal muscle resistance arterioles and to determine whether this metabolism is altered during the rapid growth phase of the rat. We attempted to quantify ATP metabolism in gastrocnemius first‐order arterioles from 8‐, 10‐, and 12‐week‐old rats. We measured ATP metabolism using an ATPase/GTPase assay with whole vessel segments as well as using a real‐time adenosine biosensor following electric field stimulation. Our first method of measuring ATP metabolism allowed us to measure the amount of free phosphate produced with ATP as a substrate. When ecto‐nucleotidase activity was inhibited by ARL67156, pyridoxal phosphate‐6‐azophenly‐2′, 4′‐disulfonic acid (PPADS), or suramin prior to adding ATP, we found that the rate of phosphate production was significantly reduced by 27%, 21%, and 22%, respectively (*P* < 0.05). Our second method of measuring ATP metabolism allowed us to measure the amount of adenosine produced following electric field stimulation of the arteriole with and without nucleotidase inhibitors. Surprisingly, we found that adenosine overflow was not attenuated by nucleotidase inhibitors. We concluded that ecto‐phosphodieterase/phyrophophatase (E‐NPP), ecto‐diadenosine polyphosphatase (ApnA), NTPDase1 and 2, and E5NT may be present on the gastrocnemius 1A arteriole and do play a role in ATP metabolism. Between the ages of 8 weeks and 12 weeks, however, overall ATP metabolism may not change.

## Abstract (precis)

The purpose of this study was to investigate the metabolism of Adenosine triphosphate (ATP) in skeletal muscle resistance arterioles and whether it is altered during the rapid growth phase of the rat. We concluded that ecto‐phosphodieterase/phyrophophatase (E‐NPP), ecto‐diadenosine polyphosphatase (ApnA), NTPDase1 and 2, and E5NT may be present on the gastrocnemius 1A arteriole and do play a role in ATP metabolism. Between the ages of 8 weeks and 12 weeks, however, overall ATP metabolism may not change.

## Introduction

Adenosine triphosphate (ATP) is a unique molecule that is involved in diverse processes in the body including muscle contraction, glucose homeostasis (Burnstock and Novak 2012), and information processing by the central nervous system (Dale et al. 2002; Gourine et al. 2005a, 2008). ATP is also increasingly important for the control of vascular tone at rest and during exercise (Buckwalter et al. 2004; Burnstock 2008a,b, 2009; Kirby et al. 2008, 2010, 2011, 2012; Crecelius et al. 2011).

Sources of ATP vary within the vasculature. The endothelium and red blood cells release ATP which can lead to vasodilation via the cAMP pathway (Ralevic and Burnstock 1998). Sympathetic nerves are another source of ATP. Vesicles on the sympathetic end terminal are known to store ATP at concentrations ranging from 1 to 200 mmol/L (Bodin and Burnstock 2001). ATP released from the nerve causes constriction by diffusing across the neurovascular synapse and activating purinergic receptors on the vascular smooth muscle (Ralevic and Burnstock 1998). ATP metabolism by enzymes present on the surface of the vascular smooth muscle and endothelium is not well understood.

ATP metabolism limits the activity of ATP‐specific receptors and enhances the activation of other purine and pyrimidine‐specific receptors (Ralevic and Burnstock 1998; Zimmermann 2000). ATP is broken down rapidly in the synapse by a variety of membrane‐bound enzymes called ecto‐nucleotidases which include, but are not limited to ecto‐ATPase, ATP diphosphohydrolase (ecto‐apyrase), ADPase, ecto 5′‐nucleotidase (E5NT), ecto‐phosphodieterase/phyrophophatase (E‐NPP), and ecto‐diadenosine polyphosphatase (ApnA). More specifically, the ecto‐nucleoside triphosphate diphosphohydrolase (E‐NTPDase) family is believed to play a large role in metabolizing extracellular ATP and ADP in the vasculature (Robson et al. 2006). NTPDase1, also known as ecto‐apyrase, is commonly found in the endothelium and vascular smooth muscle. NTPDase2, also known as ecto‐ATPase is more specific to the adventitial layer of the vasculature. NTPDase2 has a higher affinity for ATP hydrolysis versus ADP hydrolysis than NTPDase1 (Zimmermann 2000; Robson et al. 2006). Considering the location and functional properties of these E‐NTPDases, we believe NTPDase2 is primarily responsible for the metabolism of ATP released from the nerve in the neurovascular synapse of the skeletal muscle vasculature. E5NT is responsible for the majority of adenosine produced by hydrolyzing AMP (Zimmermann 2000). Each enzyme has unique characteristics and metabolizes ATP into ADP, AMP, adenosine, and/or free phosphates (Gordon 1986; Zimmermann et al. 1998), and these by‐products are commonly measured when attempting to identify nucleotidases.

Studies have shown that purinergic regulation changes during maturation and aging, and enzymatic control could contribute to these changes. Rats develop rapidly between 2 and 3 months of age which is also the transition time from puberty to young adult (Kwekel et al. 2010); it is important to keep this is in mind as this age group is considered sexually mature and is commonly used in research. Wallace et al. (2006) found both density of purinergic receptors and subtype expression change dramatically in rat tail and mesenteric arteries during this same period of time. These changes in purinergic vascular control occur before 12 weeks of age, with younger rats having a higher expression of purinergic receptors as well as a greater response to purinergic agonists than older rats (Wallace et al. 2006). Although receptor density and sensitivity are found to change, little is known about the metabolism of ATP during this time and/or whether enzymatic regulation of ATP by ecto‐nucleotidases could also be playing a role in purinergic vascular control.

The primary goal of this study was to investigate possible sources of ATP metabolism on the surface of skeletal muscle arterioles. Our secondary goal was to investigate possible changes in ATP metabolism during the rapid growth phase of the rat. In addition to these questions, we also examined changes in vascular function with ATP metabolism and expected ATP to cause greater vasoconstriction when ecto‐nucleotidases were blocked.

## Material and Methods

All protocols were submitted and approved by the Animal Care and Use Committee of the University of Arkansas, Fayetteville, Arkansas and Auburn University, Auburn, Alabama.

### General procedures

In the study, 8‐week (156 ± 3 g), 10‐week (167 ± 3 g), and 12‐week (177 ± 2 g)‐old female Fisher 344 rats were used. Rats were anesthetized with sodium pentobarbital (42.5 mg/mL), and the gastrocnemius muscles were removed from the hind limbs and stored in phosphate‐free Krebs–Ringer bicarbonate solution (NaCl 119 mmol, KCl 4.7 mmol, CaCl_2_ 2.5 mmol, MgSO_4_ 1.2 mmol, NaHCO3 25 mmol, glucose 5.5 mmol, and 0.184 g/L of glycerol in ultrapurified H_2_O, pH 7.4 at 4°C). The rats were then euthanized by pneumothorax. The first‐order (1A) arterioles were cleaned, leaving the sympathetic end terminals which are embedded in the adventitial layer intact, and removed from the lateral head of the gastrocnemius muscles. All arteriole segments were 2 mm in length. The gastrocnemius 1A arteriole was defined as the first branch of the feed artery once it entered the gastrocnemius muscle (Donato et al. 2007).

### Cannulated arterioles

Using 11‐0 ophthalmic suture, the arterioles were tied securely to micropipettes in a vessel chamber (Living Systems Inc., Burlington, VT) which was filled with Krebs‐Ringer physiological saline solution containing 1% albumin (pH 7.4, 37°C) (Pourageaud and De Mey 1998) and 2 mmol glycerol in order to optimize detection of adenosine (Llaudet et al. 2005). The bath was filled with Krebs‐Ringer physiological saline solution (pH 7.4, 37°C) and transferred to the stage of an inverted microscope (Olympus CKX41, Melville, NY). Luminal diameter was monitored during equilibration and viability testing (described below) using video calipers (Colorado video 307A Horizontal video calipers, Boulder, CO) and recorded using Chart software (AD instruments, Colorado Springs, CO). The bath was gradually warmed and maintained at 37°C for the rest of the experiment. Micropipettes were connected to independent reservoir systems. Luminal pressure was initially set at 60 cmH_2_O by elevating both reservoirs to the same level. Thirty minutes later it was raised to 90 cmH_2_O, which is similar to normal in vivo pressure in 1A arterioles (Williams and Segal 1993). The bath solution was replaced every 15 min during equilibration. Arterioles were considered viable if they constricted to phenylephrine (10^−5^ mol/L) by at least 10% and dilated by at least 20% to acetylcholine (10^−6^ mol/L).

### Series 1: Purinergic metabolism

The QuantiChrom ATPase/GTPase Assay (BioAssay Systems, Hayward, CA) was used to measure ecto‐nucleotidase activity in gastrocnemius 1A arterioles. Whole vessel segments were used to limit the contribution from intracellular nucleotidases and optimize measuring ecto‐nucleotidase activity on the surface of the arteriole. ATP (1 mmol; Sigma‐Aldrich, St. Louis, MO) was added to each vessel and left to incubate for 10 min before adding the phosphate‐specific reagent. We wished to investigate the type of ecto‐nucleotidase present on the surface of the 1A arteriole. Therefore, in a separate group of 10‐week‐old rats, we added ARL67156 (1 mmol), pyridoxal phosphate‐6‐azophenly‐2′, 4′‐disulfonic acid (PPADS, 20 *μ*mol), or suramin (2 *μ*mol) to the arteriole 20 min prior to adding ATP, the substrate. The arteriole from the opposite leg was used as the control where ATP was added without prior incubation of ARL67156, PPADS, or suramin. Therefore, the arterioles from each rat were used for one blocker and one control. ARL67156 (FPL 67156; Tocris, Bristol, U.K.) is a weak competitive inhibitor of NTPDase1 and NPP1 (Levesque et al. 2007). PPADS (Tocris, Bristol, U.K.) has a mild inhibitory effect on NTPDase1 and NTPDase2, but completely inhibits E‐NPP and ApnA (Yegutkin and Burnstock 2000). Suramin (Sigma‐Aldrich) effectively inhibits ApnA (Mateo et al. 1996). ARL67156, Suramin, and PPADS are all poor blockers for E5NT.

Phosphates can come from a variety of sources, so we investigated the specificity of the assay using an ATP analog, *α*,* β*‐methylene ATP (1 mmol, Sigma‐Aldrich). *α*,* β*‐methylene ATP is widely used as a specific P2X agonist and has the advantage of being resistant to breakdown by ecto‐nucleotidases. Therefore, if *α*,* β*‐methylene ATP is added in place of ATP, any phosphate produced would not be specific to ATP. These experiments were performed in a subset of 10‐week‐old rats. Again, using the QuantiChrom ATPase/GTPase Assay, arterioles from the same rat were used to eliminate potential interindividual differences. The arteriole from one leg received ATP and the arteriole from the other leg received *α*,* β*‐methylene ATP (1 mmol/L).

### Series 2: Adenosine overflow

Adenosine probes (Sarissa Biomedical, Coventry, U.K.) were interfaced with a MicroC potentiostat (WPI, Sarasota, FL) and a Powerlab 16/30 (AD Instruments). The probes were positioned with a micromanipulator such that the tip of the probe touched the outside wall of the arteriole. Kluess et al. (2010) provides a more detailed description of probe positioning. To account for compounds that may interfere with the adenosine signal (Gourine et al. 2005b; Llaudet et al. 2005), a null electrode (no enzyme coating) was placed in a biologically similar location to the adenosine electrode. Adenosine probes are similar to ATP probes which have been used previously to record real‐time ATP release from isolated arterioles (Kluess et al. 2010). We chose to use adenosine probes over ATP probes because ATP probes are inhibited by ecto‐nucleotidase blockers, whereas adenosine probes are not inhibited by ecto‐nucleotidase blockers. The adenosine biosensors were used previously in brain slices and are sensitive to adenosine and inosine, but they are not sensitive to ATP, UTP, or other purines (Gourine et al. 2002; Llaudet et al. 2005).

For the experiment, adenosine in concentrations ranging from 0.78 *μ*mol/L to 25 *μ*mol/L (Sigma‐Aldrich), was added to create a normal curve. Following the normal curve, the arterioles were washed five times over 15 min. The arterioles were field stimulated at 60 Hz, 32 mA for 200 impulses using a Model DS3 constant current isolated stimulator (Digitimer, Welwyn Garden City, Hertfordshire, England) and adenosine and Null signal were recorded for 1 min after the cessation of field stimulation. The arterioles were then washed (15 min) and another normal curve was created to assess loss of sensitivity in the probe over the course of the experiment. For the calculation of adenosine concentration, the normal curve shifts at the beginning and end of the experiment were averaged.

Using the adenosine probes, an additional experiment was performed to identify whether the detected adenosine overflow was originating from the breakdown of ATP. The potential enzymes that may hydrolyze ATP (or its products ADP and AMP) to adenosine include alkaline phosphatases, E5NT, and E‐NPP. Sodium orthovanadate (49 mmol), a non‐specific phosphatase inhibitor (Kumar and Corder 1980) and ARL67156 were added to the bath. Following 20 min of incubation with the antagonists, the arteriole was field stimulated at 60 Hz, 32 mA for 200 impulses and adenosine and null raw data were collected for 1 min.

Data were analyzed using Chart and Excel (Microsoft, version 14, Redmond, WA) software. Raw data from adenosine and null probes were processed by taking the integral of every 5 sec of data. Data were processed by subtracting the null probe values from the adenosine probe values and were reported as 5‐sec integrals and as a sum of all of the 5‐sec integrals for the entire minute of data collection. The resultant data were termed “overflow” because the magnitude of the purine measured is the net effect of release and metabolism or removal (Dale et al. 2002; Gourine et al. 2005a,b, 2008; Kluess et al. 2010).

### Series 3: ATP‐mediated vasoconstriction

Arterioles of 10‐week‐old rats were subjected to increasing concentrations of ATP (10^−7^ to 10^−3 ^mol/L) before and after the addition of ARL67156 (1 mmol). Constriction was monitored using video calipers interfaced with a PowerLab data acquisitions system and Chart software. The arteriole was washed and left for 15 min between each dose to prevent desensitization of the P2 receptor. Data were reported as a percent of baseline diameter.

### Statistical analysis

All summary data figures are expressed as mean ± SE. Raw data tracings of adenosine overflow are reported as the amount released from the vessel for 60 sec following electrical stimulation. Ecto‐nucleotidase activity is reported as unit of phosphate (*μ*mol) per minute.

Raw data tracings of adenosine overflow were analyzed using descriptive analyses for the pattern of release. Ecto‐nucleotidase activity was analyzed using paired student's *t*‐tests for differences in ecto‐nucleotidase activity with and without inhibitors. Differences between ecto‐nucleotidase activity with ATP versus *α*,* β*‐methylene ATP as the substrate were also found using paired student's *t*‐tests. All analyses were completed using GraphPad Prism software (GraphPad Software Inc., San Diego, CA), and the level of significance was set at *P* < 0.05 with Bonferroni's correction for multiple tests.

## Results

### Series 1: Purinergic metabolism

**Figure 1.**
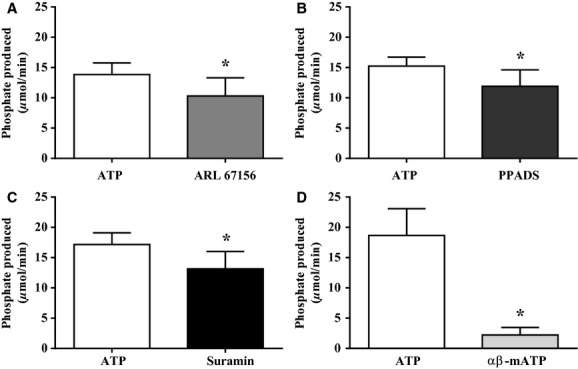
The summary of the ecto‐nucleotidase activity with and without blockers (*n* = 3 for each group). The amount of phosphate produced by an arteriole was also significantly reduced when in the presence of ARL67156 (A), PPADS (B), and suramin (C) before the addition of ATP compared to those in only ATP. Arterioles with *α*,* β*‐methylene ATP as the substrate produced significantly less phosphate than those that used ATP (D). Asterisks (*) represent significantly less phosphate produced per min (*P* < 0.05).

**Figure 2.**
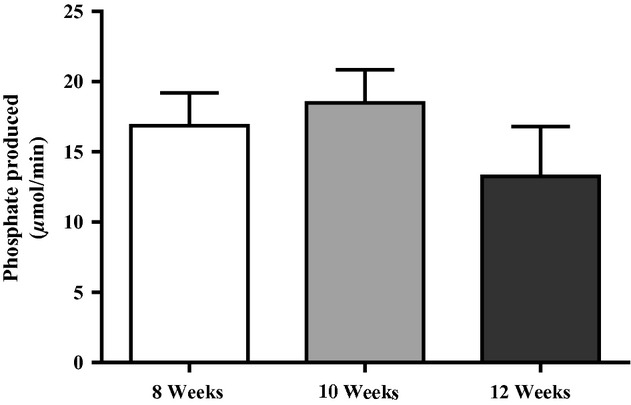
The summary of phosphate produced per minute in arterioles from 8‐, 10‐, and 12‐week‐old rats. There were no differences in the amount of phosphate produced among the age groups.

Arterioles which were incubated in ARL67156 (Fig. 1A) prior to the addition of ATP resulted in a 26 ± 8% decrease in phosphate production (*n* = 3, *P* < 0.05). PPADS (Fig. 1B) caused a 21 ± 11% decrease in phosphate production (*n* = 3, *P* < 0.05), and suramin (Fig. 1C) caused a 22 ± 10% decrease in phosphate produced per minute (*n* = 3, *P* < 0.05). Arterioles in *α*,* β*‐methylene ATP (Fig. 1D) reduced the amount of phosphate produced by 85 ± 15% compared to those in ATP (*n* = 3, *P* < 0.05). All ecto‐nucleotidase inhibitors significantly attenuated the production of free phosphate, and the majority of phosphate produced was from the breakdown of ATP. Figure 2 is the summary data for the metabolism of ATP in arterioles from 8‐week (*n* = 6), 10‐week (*n* = 6), and 12‐week‐old (*n* = 5) rats. There were no significant differences in ecto‐nucleotidase activity among the age groups (*P* > 0.05). Vessel diameter was significantly different from 8 weeks to 10 weeks, but diameter at 12 weeks was not different from that at either 8 weeks or 10 weeks (8 weeks: 249 ± 10 *μ*m, 10 weeks: 304 ± 14 *μ*m, 12 weeks: 282 ± 12 *μ*m).

### Series 2: Adenosine overflow

**Figure 3.**
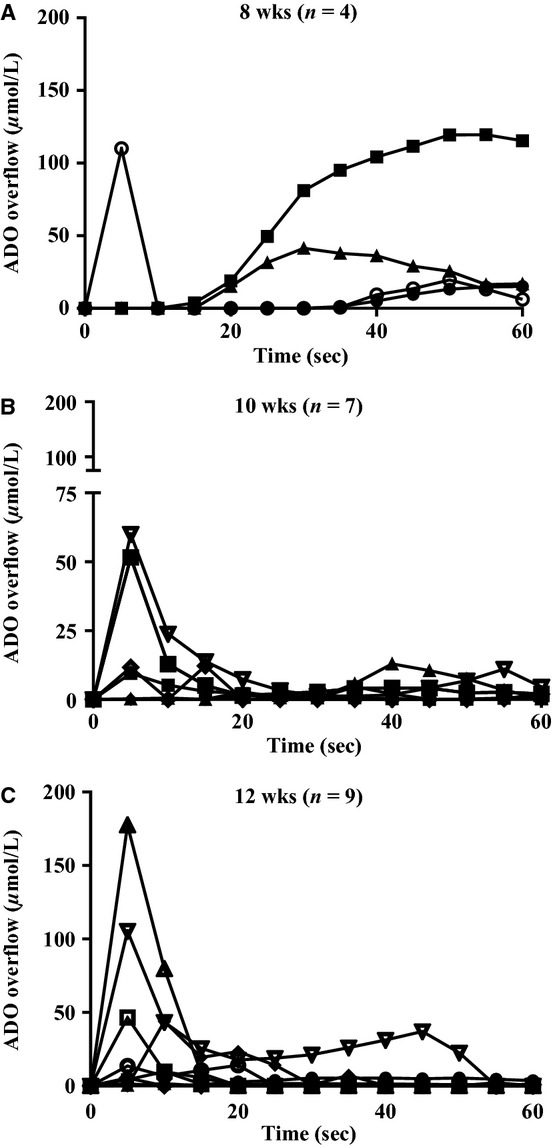
Adenosine overflow (ADO) following field stimulation of arterioles. Each line represents the adenosine overflow from one rat. Adenosine overflow was relatively similar among 8 (A)‐, 10 (B)‐, and 12 (C)‐week‐old rats where the greatest amount of adenosine was detected in the first 20 sec following stimulation.

Adenosine overflow was measured in the arterioles from 8‐week (*n* = 6), 10‐week (*n* = 9), and 12‐week‐old (*n* = 10) rats (Fig. 3). Each line on Figure 3 represents the data from a single arteriole. In the 8‐week‐old rats (Fig. 3A), the range of summed integrals was 0–250 *μ*mol/L adenosine (two arterioles produced very little to no adenosine overflow). In arterioles from the 10‐week‐old rats (Fig. 3B), the range of summed integrals was 0.62–139 *μ*mol/L adenosine. Of these rats, seven arterioles produced an appreciable amount of adenosine and are presented on the graph (Fig. 3B). In the 12‐week‐old rats (Fig. 3C), the range of summed integrals was 0–349 *μ*mol/L adenosine with one rat showing no detectible adenosine. The majority of arterioles did produce adenosine when electrically stimulated.

**Figure 4.**
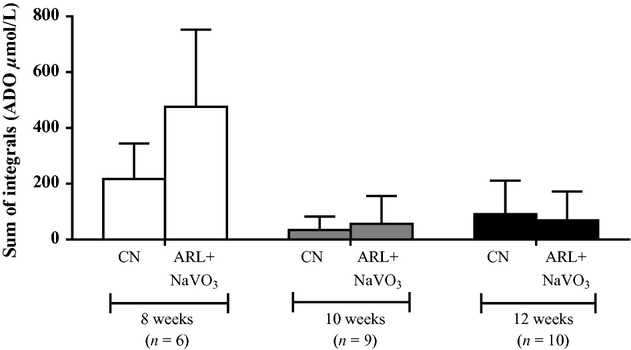
The summary of the adenosine (ADO) overflow during the control condition (CN) and following the addition of the ecto‐nucleotidase antagonist, ARL67156 (ARL) and the phosphatase inhibitor, sodium orthovanadate (NaVO_3_). The inhibitors had no effect on adenosine overflow at 8 weeks, 10 weeks, or 12 weeks of age.

In order to identify whether ecto‐nucleotidase caused the breakdown of ATP, we also recorded adenosine overflow after incubating the arterioles in both ARL67156 and sodium orthovanadate (Fig. 4A). These data are reported as an average of the sum of the integrals over 1 min of recording. In all age groups (8 weeks: *n* = 6; 10 weeks: *n* = 9; and 12 weeks: *n* = 10), ARL67156 and sodium orthovanadate together failed to reduce adenosine production. Therefore, the adenosine produced may not be directly from the metabolism of ATP.

### Series 3: ATP‐mediated vasoconstriction

**Figure 5.**
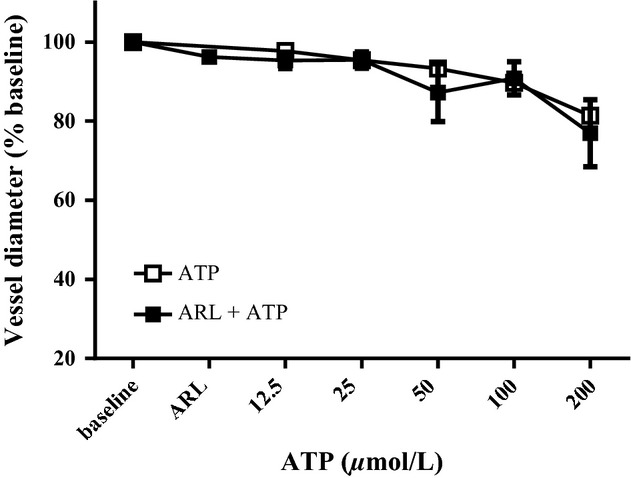
Vascular responsiveness to increasing concentrations of ATP before and after ARL67156. ARL67156 did not increase vasoconstriction to ATP.

ATP concentration response curves were performed in arterioles from 10‐week‐old (*n* = 4)‐rats (Fig. 5) before and after the addition of ARL67156. ATP (200 *μ*mol/L) caused significant constriction compared to baseline diameter with ARL67156 (77 ± 8%) and without (81 ± 7%). ARL67156 did not allow significantly greater vasoconstriction to increasing concentrations of ATP.

## Discussion

The purpose of this study was to investigate the metabolism of ATP in skeletal muscle resistance arterioles and to determine whether this metabolism is altered during the rapid growth phase of the rat. We found that the ecto‐nucleotidase inhibitors ARL67156, PPADS, and suramin individually reduced ATP hydrolysis indicating that the E‐NPP family as well as other nucleotidases may be present on the resistance arteriole. In similar resistance arterioles, the overall rate of ATP hydrolysis did not change between 8 and 12 weeks of age. With regard to vascular function, we expected the resistance arterioles to be more sensitive to ATP when ecto‐nucleotidases were inhibited than when they were not. However, we found that ATP hydrolysis in the presence of ARL67156 did not increase the vasoconstriction response to ATP.

### Purinergic metabolism

One of the main purposes in this study was to measure membrane‐bound ecto‐nucleotidase activity on the surface of gastrocnemius 1A arterioles. ATP hydrolysis is typically measured using high performance liquid chromatography (Yagi et al. 1991; Westfall et al. 2000), but this method necessitates grinding of the arteriole which would eliminate the ability to determine membrane‐bound ecto‐nucleotidase activity from other sites of nucleotidases activity. Phosphate is a first‐order product of ATP hydrolysis by E‐NTPDase 1, E‐NTPDase 2, E‐NPP, alkaline phosphatase, and E5NT. Using several antagonists, we measured ecto‐nucleotidase activity on the surface of the gastrocnemius 1A arteriole. We found that individually ARL67156, PPADS, and suramin all attenuated phosphate production. Suramin is a strong blocker for the ApnA and E‐NPP family of ecto‐nucleotidases at the concentration used (Stefan et al. 2006). The ecto‐nucleotidases inhibitors ARL67156 and PPADS are also strong inhibitors of ApnA and E‐NPP. These data suggest that the E‐NPP family plays a significant role in ATP hydrolysis. This is not surprising since members of the E‐NPP family are widely distributed throughout the body (Stefan et al. 2006).

Previous work has shown that ecto‐nucleotidase activity varies dramatically in the rat hippocampus and caudate nucleus during development and maturation from about 2 weeks to 1 year of age (Banjac et al. 2001). The study found that between 18 days and 30 days of age ecto‐ATPase activity almost doubled. Then, between 30 days and 90 days, ecto‐ATPase activity returned to a similar rate seen at 18 days. From 90 days on, ecto‐ATPase activity slowly increased again, but the fluctuation of activity was not nearly as large in magnitude as it was in the first 90 days (Banjac et al. 2001). According to Banjac et al. (2001) and Wallace et al. (2006) ecto‐nucleotidase activity may differ among age groups in rats younger than 8 weeks old. We chose to study 2–3‐month‐old rats because this is a common age range for vascular studies, and there has been some concern regarding the vascular changes with maturation in this age range. Our data suggest that ecto‐nucleotidase activity in skeletal muscle resistance arterioles is relatively constant between the ages of 8 weeks and 12 weeks.

Alternately, we measured adenosine release as an indicator of ATP hydrolysis. Specifically, the E‐NPP, the E5NT, and the alkaline phosphatase families of ecto‐nucleotidases can produce adenosine as a by‐product. We found that the ecto‐nucleotidase inhibitors ARL67156 and sodium orthovanadate did not attenuate adenosine production and nearly increased adenosine production in the 8‐week‐old group. The increase in adenosine may, in part, be caused by the secondary vasoconstriction that occurs with ARL67156 and sodium orthovanadate. For the 10 and 12 week groups, the adenosine measured was at the bottom of the detectible range by the biosensors; therefore, it was difficult to measure an attenuation of adenosine in these age groups. A limitation of using adenosine to estimate ATP hydrolysis is that adenosine is the third or fourth step from ATP (following ADP and AMP) in many ecto‐nucleotidase reactions and is only created by E‐NPP, the E5NT, and the alkaline phosphatase families of ecto‐nucleotidases (Zimmermann 2000).

### Adenosine overflow

To our knowledge, this is the first time that real‐time adenosine/inosine measurements were made in an isolated arteriole following field stimulation. Arterioles from 8‐week‐old animals had the most adenosine detected between 20 and 60 sec after field stimulation. In 10‐week and 12‐week‐old rats, the most adenosine detected was in the first 20 sec following stimulation; whereas little adenosine was detected between 20 sec and 60 sec following stimulation. The first 20 sec following stimulation is noteworthy because that is the time period where the nerve releases ATP (Todorov et al. 1996). Therefore, the early peak of adenosine in the 10‐ and 12‐week‐old rats may be from the breakdown of ATP released from the nerve. We were unable to attenuate adenosine overflow using the available ecto‐nucleotidase inhibitors. ARL67156 is a weak inhibitor of NTPDase1 and NPP1 and is not an effective inhibitor of NTPDase2 or E5NT (Levesque et al. 2007). Therefore, it is likely that NTPDase2 and/or E5NT are the enzymes responsible for ATP metabolism following field stimulation of the arteriole. The phosphate assay allowed more direct measurement of NTPDase2 activity, whereas the adenosine biosensor probes measured more E5NT activity. ADP and AMP hydrolysis also produce adenosine along with the metabolism of ATP. The metabolism of adenosine by adenosine deaminase produces inosine which is also detected by the biosensor probe. Measuring adenosine to estimate ATP metabolism is somewhat limited by the availability of specific ecto‐nucleotidase inhibitors; however, the idea of using adenosine probes to measure ecto‐nucleotidase activity should be considered in future studies.

### Limitations

While overall ecto‐nucleotidase activity did not appear to change from 8 weeks to 12 weeks, we cannot say whether specific ecto‐nucleotidases changed during this time; we only measured changed in ecto‐nucleotidase activity as a whole. Also, we only used ecto‐nucleotidase inhibitors individually and not in combination with one another when using the ATPase/GTPase assay. Our sample size for the blocking studies was very small and this should be considered. In future studies, combining inhibitors may allow one to check for redundancy in the ATP metabolism. One could also eliminate the possibility of ecto‐nucleotidase activity coming from the endothelium by removing the endothelium and then running the ATPase/GTPase assay. Another limitation of this study was the lack of specific ecto‐nucleotidase inhibitors. Most ecto‐nucleotidase inhibitors are broad antagonists with the only real exception being ARL67156.

In conclusion, our findings suggest that several different ecto‐nucleotidases are present on the gastrocnemius 1A arteriole including E‐NPP, ApnA, NTPDase 1, and NTPDase 2, and E5NT. Surprisingly, ecto‐nucleotidase activity in the 1A arteriole is not altered during the 8–12‐week rapid growth period of the rat, but this may still occur in rats younger than 8 weeks. Vascular responsiveness to the addition of ATP is not increased in the presence of ecto‐nucleotidase inhibitor ARL67156; however, this is understandable considering that ARL67156 attenuated but did not abolish ATP hydrolysis. Differences in ecto‐nucleotidase families present on tissue and activity of these enzymes may, in part, explain some of the differences in purine actions across different animals and in different vascular beds. Hopefully, there will be development of more specific antagonists for ecto‐nucleotidase families, so we can better understand the role that ecto‐nucleotidases play in vascular function.

## Acknowledgments

The authors thank Ryan Bailey and Konrad Siemek for their technical assistance during the project.

## Conflict of Interest

None declared.

## References

[b1] Banjac A. Nedeljkovic N. Horvat A. Kanazir D. Nikezic G. 2001 Ontogenetic profile of ecto‐ATPase activity in rat hippocampal and caudate nucleus synaptic plasma membrane fractions. Physiol. Res.; 50:411-417 11551148

[b2] Bodin P. Burnstock G. 2001 Purinergic signalling: ATP release. Neurochem. Res.; 26:959-969 11699948 10.1023/a:1012388618693

[b3] Buckwalter J. B. Taylor J. C. Hamann J. J. Clifford P. S. 2004 Do P2X purinergic receptors regulate skeletal muscle blood flow during exercise? Am. J. Physiol. Heart Circ. Physiol.; 286:H633-H639 14551053 10.1152/ajpheart.00572.2003

[b4] Burnstock G. 2008a Dual control of vascular tone and remodelling by ATP released from nerves and endothelial cells. Pharmacol. Rep.; 60:12-20 18276981

[b5] Burnstock G. 2008b Endothelium‐derived vasoconstriction by purines and pyrimidines. Circ. Res.; 103:1056-1057 18988902 10.1161/CIRCRESAHA.108.187963

[b6] Burnstock G. 2009 Purinergic signalling: past, present and future. Braz. J. Med. Biol. Res.; 42:3-8 18853040 10.1590/s0100-879x2008005000037

[b7] Burnstock G. Novak I. 2012 Purinergic signalling in the pancreas in health and disease. J. Endocrinol.; 213:123-141 22396456 10.1530/JOE-11-0434

[b8] Crecelius A. R. Kirby B. S. Richards J. C. Garcia L. J. Voyles W. F. Larson D. G. 2011 Mechanisms of ATP‐mediated vasodilation in humans: modest role for nitric oxide and vasodilating prostaglandins. Am. J. Physiol. Heart Circ. Physiol.; 301:H1302-H1310 21784984 10.1152/ajpheart.00469.2011 PMC3197353

[b9] Dale N. Gourine A. V. Llaudet E. Bulmer D. Thomas T. Spyer K. M. 2002 Rapid adenosine release in the nucleus tractus solitarii during defence response in rats: real‐time measurement in vivo. J. Physiol.; 544:149-160 12356888 10.1113/jphysiol.2002.024158 PMC2290567

[b10] Donato A. J. Lesniewski L. A. Delp M. D. 2007 Ageing and exercise training alter adrenergic vasomotor responses of rat skeletal muscle arterioles. J. Physiol.; 579:115-125 17082231 10.1113/jphysiol.2006.120055 PMC2075385

[b11] Gordon J. L. 1986 Extracellular ATP: effects, sources and fate. Biochem. J.; 233:309-319 3006665 10.1042/bj2330309 PMC1153029

[b12] Gourine A. V. Llaudet E. Thomas T. Dale N. Spyer K. M. 2002 Adenosine release in nucleus tractus solitarii does not appear to mediate hypoxia‐induced respiratory depression in rats. J. Physiol.; 544:161-170 12356889 10.1113/jphysiol.2002.024174 PMC2290570

[b13] Gourine A. V. Llaudet E. Dale N. Spyer K. M. 2005a ATP is a mediator of chemosensory transduction in the central nervous system. Nature; 436:108-111 16001070 10.1038/nature03690

[b14] Gourine A. V. Llaudet E. Dale N. Spyer K. M. 2005b Release of ATP in the ventral medulla during hypoxia in rats: role in hypoxic ventilatory response. J. Neurosci.; 25:1211-1218 15689558 10.1523/JNEUROSCI.3763-04.2005 PMC6725960

[b15] Gourine A. V. Dale N. Korsak A. Llaudet E. Tian F. Huckstepp R. 2008 Release of ATP and glutamate in the nucleus tractus solitarii mediate pulmonary stretch receptor (Breuer‐Hering) reflex pathway. J. Physiol.; 586:3963-3978 18617567 10.1113/jphysiol.2008.154567 PMC2538935

[b16] Kirby B. S. Voyles W. F. Carlson R. E. Dinenno F. A. 2008 Graded sympatholytic effect of exogenous ATP on postjunctional alpha‐adrenergic vasoconstriction in the human forearm: implications for vascular control in contracting muscle. J. Physiol.; 586:4305-4316 18617568 10.1113/jphysiol.2008.154252 PMC2652174

[b17] Kirby B. S. Crecelius A. R. Voyles W. F. Dinenno F. A. 2010 Vasodilatory responsiveness to adenosine triphosphate in ageing humans. J. Physiol.; 588:4017-4027 20807789 10.1113/jphysiol.2010.197814 PMC3000589

[b18] Kirby B. S. Crecelius A. R. Voyles W. F. Dinenno F. A. 2011 Modulation of postjunctional alpha‐adrenergic vasoconstriction during exercise and exogenous ATP infusions in ageing humans. J. Physiol.; 589:2641-2653 21486772 10.1113/jphysiol.2010.204081 PMC3115831

[b19] Kirby B. S. Crecelius A. R. Voyles W. F. Dinenno F. A. 2012 Impaired skeletal muscle blood flow control with advancing age in humans: attenuated ATP release and local vasodilation during erythrocyte deoxygenation. Circ. Res.; 111:220-230 22647875 10.1161/CIRCRESAHA.112.269571 PMC3393524

[b20] Kluess H. A. Stone A. J. Evanson K. W. 2010 ATP overflow in skeletal muscle 1A arterioles. J. Physiol.; 588:3089-3100 20566660 10.1113/jphysiol.2010.193094 PMC2956947

[b21] Kumar A. Corder C. N. 1980 Diuretic and vasoconstrictor effects of sodium orthovanadate on the isolated perfused rat kidney. J. Pharmacol. Exp. Ther.; 213:85-90 6244393

[b22] Kwekel J. C. Desai V. G. Moland C. L. Branham W. S. Fuscoe J. C. 2010 Age and sex dependent changes in liver gene expression during the life cycle of the rat. BMC Genomics; 11:675 21118493 10.1186/1471-2164-11-675 PMC3012673

[b23] Levesque S. A. Lavoie E. G. Lecka J. Bigonnesse F. Sevigny J. 2007 Specificity of the ecto‐ATPase inhibitor ARL 67156 on human and mouse ectonucleotidases. Br. J. Pharmacol.; 152:141-150 17603550 10.1038/sj.bjp.0707361 PMC1978278

[b24] Llaudet E. Hatz S. Droniou M. Dale N. 2005 Microelectrode biosensor for real‐time measurement of ATP in biological tissue. Anal. Chem.; 77:3267-3273 15889918 10.1021/ac048106q

[b25] Mateo J. Rotllan P. Miras‐Portugal M. T. 1996 Suramin–a powerful inhibitor of neural ecto‐diadenosine polyphosphate hydrolase. Br. J. Pharmacol.; 119:1-2 8872348 10.1111/j.1476-5381.1996.tb15668.x PMC1915732

[b26] Pourageaud F. De Mey J. G. 1998 Vasomotor responses in chronically hyperperfused and hypoperfused rat mesenteric arteries. Am. J. Physiol.; 274:H1301-H1307 9575935 10.1152/ajpheart.1998.274.4.H1301

[b27] Ralevic V. Burnstock G. 1998 Receptors for purines and pyrimidines. Pharmacol. Rev.; 50:413-492 9755289

[b28] Robson S. C. Sevigny J. Zimmermann H. 2006 The E‐NTPDase family of ectonucleotidases: structure function relationships and pathophysiological significance. Purinergic Signal.; 2:409-430 18404480 10.1007/s11302-006-9003-5 PMC2254478

[b29] Stefan C. Jansen S. Bollen M. 2006 Modulation of purinergic signaling by NPP‐type ectophosphodiesterases. Purinergic Signal.; 2:361-370 18404476 10.1007/s11302-005-5303-4 PMC2254485

[b30] Todorov L. D. Mihaylova‐Todorova S. Craviso G. L. Bjur R. A. Westfall D. P. 1996 Evidence for the differential release of the cotransmitters ATP and noradrenaline from sympathetic nerves of the guinea‐pig vas deferens. J. Physiol.; 496Pt. 3:731-748 8930840 10.1113/jphysiol.1996.sp021723 PMC1160860

[b31] Wallace A. Knight G. E. Cowen T. Burnstock G. 2006 Changes in purinergic signalling in developing and ageing rat tail artery: importance for temperature control. Neuropharmacology; 50:191-208 16226282 10.1016/j.neuropharm.2005.08.019

[b32] Westfall T. D. Menzies J. R. Liberman R. Waterston S. Ramphir N. Westfall D. P. 2000 Release of a soluble ATPase from the rabbit isolated vas deferens during nerve stimulation. Br. J. Pharmacol.; 131:909-914 11053210 10.1038/sj.bjp.0703662 PMC1572418

[b33] Williams D. A. Segal S. S. 1993 Feed artery role in blood flow control to rat hindlimb skeletal muscles. J. Physiol.; 463:631-646 8246199 10.1113/jphysiol.1993.sp019614 PMC1175363

[b34] Yagi K. Shinbo M. Hashizume M. Shimba L. S. Kurimura S. Miura Y. 1991 ATP diphosphohydrolase is responsible for ecto‐ATPase and ecto‐ADPase activities in bovine aorta endothelial and smooth muscle cells. Biochem. Biophys. Res. Commun.; 180:1200-1206 1835387 10.1016/s0006-291x(05)81323-3

[b35] Yegutkin G. G. Burnstock G. 2000 Inhibitory effects of some purinergic agents on ecto‐ATPase activity and pattern of stepwise ATP hydrolysis in rat liver plasma membranes. Biochem. Biophys. Acta; 1466:234-244 10825445 10.1016/s0005-2736(00)00165-6

[b36] Zimmermann H. 2000 Extracellular metabolism of ATP and other nucleotides. Naunyn Schmiedebergs Arch. Pharmacol.; 362:299-309 11111825 10.1007/s002100000309

[b37] Zimmermann H. Braun N. Kegel B. Heine P. 1998 New insights into molecular structure and function of ectonucleotidases in the nervous system. Neurochem. Int.; 32:421-425 9676740 10.1016/s0197-0186(97)00126-5

